# Stem Cells in Post-Stroke Regenerative Therapy: Current Role of Wharton’s Jelly Mesenchymal Stem Cells in the Orchestrum

**DOI:** 10.3390/brainsci16080775

**Published:** 2026-07-23

**Authors:** Anastassiya Ganina, Naizabek Yerzhigit, Oleg Lookin, Aliya Orassay, Galiya Shaimardanova, Elmira Chuvakova, Manarbek Askarov, Abay Baigenzhin

**Affiliations:** JSC National Scientific Medical Center, 42 Abylay Khan Ave., Astana 010009, Kazakhstan; naizabek1997@gmail.com (N.Y.); lookinoleg@gmail.com (O.L.); aliyaorassay@gmail.com (A.O.); galiya_masugut@mail.ru (G.S.); e.chuvakova@nnmc.kz (E.C.); illak@mail.ru (M.A.); a.baigenzhin@nnmc.kz (A.B.)

**Keywords:** stroke, neuronal cells, Wharton’s jelly, mesenchymal stem cells, secretome, exosomes, lyophilization, cell-free biomedical products

## Abstract

**Highlights:**

**What are the main findings?**

- Regenerative potential of mesenchymal stem cell (MSC) therapies, in particular using Wharton’s jelly MSCs, is supported by numerous preclinical studies and clinical trials.
- Therapies that use cell-free products like secretome and exosomes represent relatively new but promising approaches to recovery post-stroke states at the molecular and cellular level with minimal adverse effects.

**What are the implications of the main findings?**

- Post-stroke rehabilitation needs complex treatments that may require antithrombotic and endovascular drugs, physiotherapy, and digital technology-based approaches, often in combination and during prolonged periods.
- The cell-free therapies that use extracellular vesicles and exosomes secreted by Wharton’s jelly MSCs can be considered an effective adjunct therapy in selected stroke patients, especially after ischemic stroke.

**Abstract:**

Background/Objectives: Modern approaches for post-stroke rehabilitation cover mechanistically different ways—from physiotherapy to digital technologies. Among these approaches, stem cell-based therapy represents probably the most complex but promising strategy. Methods: We discuss the current state-of-the-art of using mesenchymal stem cells (MSCs) in post-stroke regenerative therapy. Despite relatively wide use of bone marrow and adipose tissue MSCs, these cells represent a more mature (“adult”) state, which limits their proliferative and regenerative potentials. Compared to the “adult” MSCs, less “mature” MSCs obtained from umbilical cord, specifically Wharton’s jelly MSCs (WJ-MSCs), demonstrate unique functional capabilities and are free from certain technical and ethical issues. Results: The molecular and cellular mechanisms of action of WJ-MSCs are thoroughly discussed in comparison with abundantly used “adult” types of MSCs. We also comparatively evaluate their preclinical and clinical application for treating post-stroke patients. Recent findings indicate that not only MSCs but also their secretome/exosomes (cell-free product) represent a therapeutically beneficial cellular drug in post-stroke recovery. Specially designed and carefully evaluated protocols, which preserve the bioactivity of the cell-free product intact, are mentioned. Neuroprotective and neuroreparative properties of cell-free products—secretome and exosomes—derived from Wharton’s jelly MSCs are summarized. Conclusions: Cell-free products obtained from WJ-MSCs are an innovative adjunct therapy for post-stroke disorders, despite certain challenges and limitations of this type of therapy still present. By further investigation of the molecular composition and biological mechanisms of the WJ-MSC secretome and exosomes, their clinical applicability in neuroinflammatory and neurodegenerative pathologies will be promoted.

## 1. Brief Overview of Currently Used Therapies in Post-Stroke Rehabilitation

Ischemic stroke is the most common type of stroke in the world, with over 65% of all types of strokes, followed by hemorrhagic stroke with nearly 30% of global cases, which significantly affects the health and economic status of the global population, especially the elderly [1]. Currently, emergency therapy primarily relies on specific drugs for thrombolysis and endovascular therapy in the early stages of the disease, as well as mechanical thrombectomy, which are critical steps for reducing brain tissue damage in acute hypoxia [2,3,4,5,6,7]. Despite these methods having improved the timeliness and effectiveness of treatment, many challenges remain to be overcome, particularly in optimizing treatment strategies and personalizing therapy for post-stroke recovery.

The implementation of early interventions after stroke (e.g., endovascular thrombectomy) is of immense importance for optimal recovery because any 10 min increase in the initiation of the recovery procedure may result in the deterioration of the ability for full recovery [4,8,9]. The initial (acute) phase of stroke rehabilitation may last up to one week after the stroke with the use of specific medications that provide antithrombotic effects, facilitate the recovery of metabolism in hypoxia-affected cells, and smooth the inflammatory processes triggered by apoptosis and necrosis of terminally damaged neuronal cells [10,11,12,13]. In some patients with a compromised immune system or pre-existing comorbidities, the post-stroke immune activation may be associated with systemic immune reactivity, which greatly reduces the recovery capability in these patients [14].

Physical rehabilitation activities are recommended within the first 24–72 h after the stroke to facilitate improving motor functions, accelerating the recovery of self-care abilities, and reducing the risk of permanent disability [15]. This is also associated with the activation of brain neuroplasticity, the formation of new neural connections, and the stimulation of restorative processes [16,17]. However, very early resume for physical activity may induce adverse effects in some patients, as shown by comparing death rates in those with early post-stroke mobilization and without it [18,19,20]. Current clinical guidelines, e.g., by the American Heart Association/American Stroke Association, therefore do not recommend physical mobilization within the 24 h post-stroke [21].

The so-called subacute phase starts from one week after the stroke and may last up to 6 months; this is the most appropriate phase for treating the patient by approaches other than the use of antithrombotic and endovascular medications [3,7,22,23]. It is often reported that the recovery (either full or partial) of motor, sensory, and cognitive functions occurs within a 3-to-6 month time window [24,25,26]. More prolonged intervals, e.g., one year or more from the stroke episode, are generally not considered suitable for rehabilitation because the damaged brain tissue and affected locomotor neurons may already have been pathologically remodeled [27,28,29]. However, some studies demonstrate that even beyond the subacute phase, e.g., later than one year post-stroke, the recovery can occur in certain patients [30]. Of note, distinct features can restore with different rates, and some features may be restored within a time window and stop recovery beyond this window, while other features continue to recover over a longer period [24]. It has also been shown that movement features do not follow the same recovery pattern and may show non-linear or peaked behavior [26], especially if the extent of physical exercise is increased [31].

**Figure 1:**
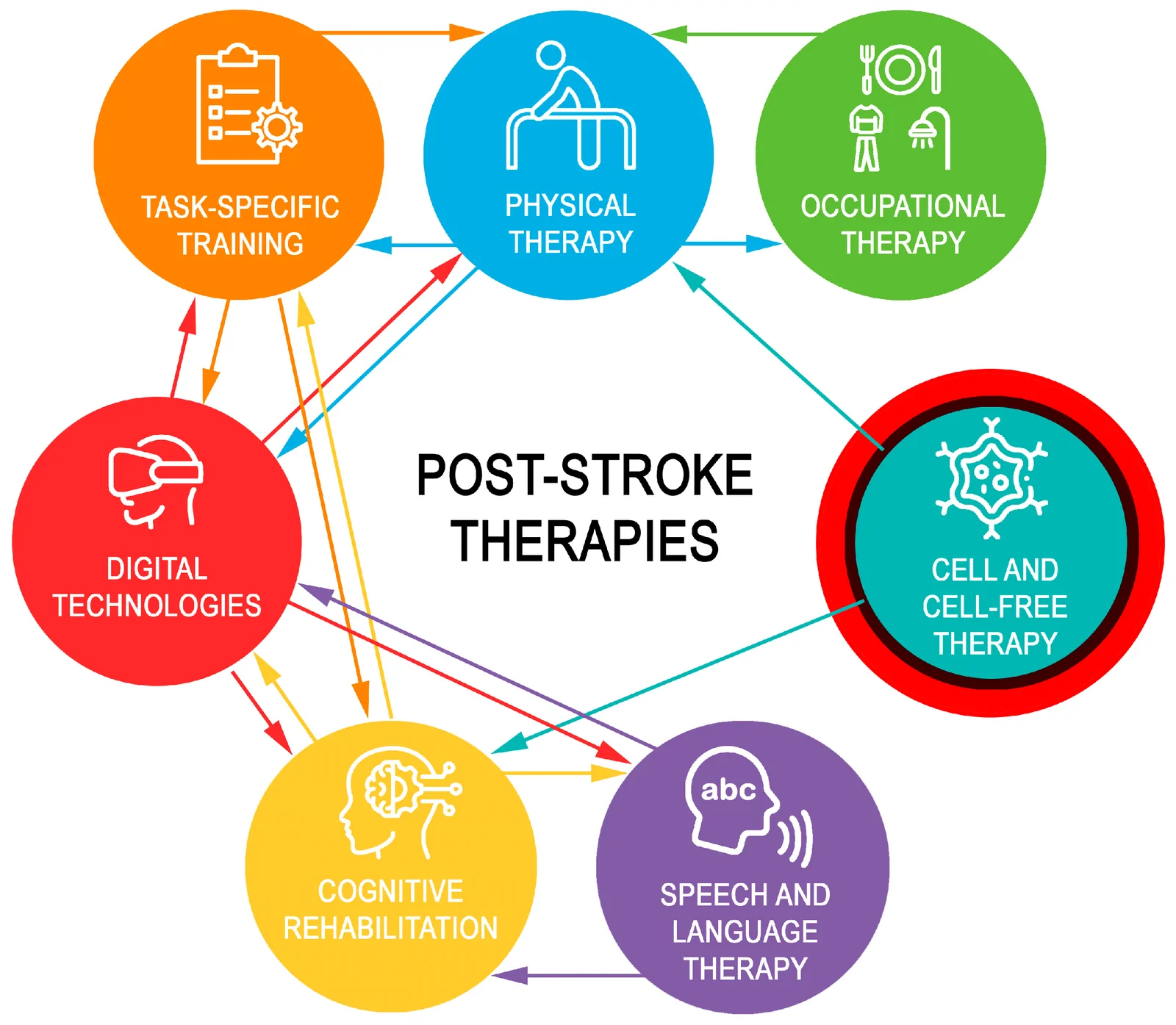
Most common types of post-stroke rehabilitation. Certain types of rehabilitation may have interconnections/interrelations as shown by the arrows. The “multistrategic therapeutic space” may even have smoother interdependencies between different types of therapies. Cell therapies are discussed in this review in detail; the corresponding type of stroke therapy is highlighted in the figure by a red band around the circle.

Over the past decade, the idea of a multidisciplinary approach to post-stroke care has grown significantly, encompassing not only medication, rehabilitation, and physical exercises but also long-term care, psychological support, risk factor control, nutritional therapy, and the implementation of digital technologies [32,33,34]. Moreover, an individualized approach that takes into account the type of stroke, severity of the damage, and comorbidities was found to be a primary point of rehabilitation [15]. As a variety of post-stroke therapies exist with certain interconnections/interdependencies between some of them (Figure 1), a personalized rehabilitation plan must be developed by an interdisciplinary approach, including neurologists, physiotherapists, occupational therapists, speech therapists, and other healthcare professionals [35,36].

For example, physiotherapy interventions, such as Bobath techniques, proprioceptive neuromuscular facilitation, constraint-induced movement therapy, and electrical stimulation, are often used to restore the impaired mobility [37,38,39,40,41]. Gait training, strength-building exercises, and balance exercises organized by physiotherapists play a key role in enhancing motor function [42,43]. Importantly, the rehabilitation of cognitive function by regular exercise is currently considered a contributing component of the global rehabilitation plan [44]. These personalized exercises and techniques actively contribute to correcting gait abnormalities. In addition, as the pathophysiology of the post-stroke changes is dependent on the development of inflammation in affected tissues, anti-inflammatory treatments are important to reduce the outcomes of ischemic stroke [45]. However, despite the intuitive benefits of various post-stroke rehabilitation treatments, heterogeneity of reported methods, differences in protocols, small sample sizes, and a limited number of studies focused on patients with hemorrhagic stroke altogether limit their wide use. Questions regarding the safety, the timing of rehabilitation initiation, and the intervention intensity remain unresolved, especially for patients with intracranial hemorrhage [46,47].

Digital technologies are another key component of rehabilitation as they provide computerized techniques, robotic-assisted devices, and telerehabilitation approaches [48,49]. Nowadays, further incorporation of modern technologies such as mobile applications, virtual reality (VR), artificial intelligence (AI), assistive devices, intercommunication between smart devices (also known as ‘Internet of Things’), and adaptive computer-based strategies has opened up new opportunities for personalized and effective post-stroke therapy [34]. The evolving landscape of rehabilitation technologies includes the implementation of transcranial direct current stimulation in combination with cognitive rehabilitation and rehabilitation of upper limbs, VR, and brain–computer interfaces, often assisted by AI-based tools [38,50,51].

Finally, one of the most promising approaches—and mechanistically differing from physical activity or digital technologies—is stem cell-based therapy. It is well known that stem cells of various origins (embryonic stem cells, ESCs; induced pluripotent stem cells, iPSCs; adult stem cells like mesenchymal stem cells, MSCs) are widely used as therapeutic material in regenerative medicine, anti-immune reactivity, reparation of damaged organs and tissues, and cancer treatment. Our review aims to discuss the benefits and limitations of MSC-based approaches, with specific focus on Wharton’s jelly MSCs (WJ-MSCs) as one of the most powerful types of stem cells. Here, we first briefly discuss the main molecular and cellular mechanisms that underlie the physiopathology of post-stroke neuronal damage. Next, we discuss how “adult” stem cells (from bone marrow and adipose tissue) can repair or modulate the affected mechanism to prevent further cell damage and switch to their regeneration. In the final sections, we delve deeper into a “young” type of MSC—WJ-MSCs—where we discuss available results on efficacy of live stem cells and their exosomes/secretome in treating stroke-induced damage of brain neuronal tissue. We summarize key differences between “young” and “adult” MSCs in terms of their physiological roles and cell mechanisms to highlight a promising potential of WJ-MSCs in post-stroke therapy.

## 2. Molecular and Cellular Mechanisms of Post-Stroke Damage of Neuronal Tissue

The post-stroke rehabilitation implies the recovery of a plethora of certain damaged/impaired cell functions. Several molecular processes accompany stroke-related cell injury and death: excitotoxicity, mitochondrial dysfunction, abnormal protein folding, oxidative stress, autophagy, and inflammatory responses [11,52,53,54,55,56]. Accordingly, multiple pathways are activated, including apoptotic, autophagy, pyroptosis, ferroptosis, and necrosis signaling [57]. During stroke, damaged neurons release cellular factors, e.g., damage-associated molecular patterns (DAMPs), fractalkine (also known as CX3CL1), and calcium-binding proteins, which are involved in activation of microglia and chemoattraction of T cells to the damaged region [58,59,60,61,62]. The signaling cascades triggered by dying neuronal cells also participate in molecular communications between oligodendrocytes, astrocytes, and microglia [63,64]. Secondary to ischemia and cell necrosis, an inflammatory response develops, which is associated with microglia activation, tissue infiltration by neutrophils and macrophages from the blood, and damage to the blood–brain barrier [64]. This process is largely driven by toxic inflammatory mediators, including interleukin-1β (IL-1β), interleukin-6 (IL-6), and tumor necrosis factor α (TNF-α) [65,66,67]. One of the contributors to this mechanism is astrocytes. Upon the onset of irreversible damage of neuronal cells and myelin destruction, astrocytes start digesting myelin debris, activate the Nuclear Factor Kappa-Light-Chain-Enhancer of Activated B Cells (NF-κB)-mediated pathway, and finally release chemokines participating in the activation of immune response [63,68].

The lack or shortage of oxygen and nutrient supply during occlusion of vessels in brain tissue is strongly affecting the metabolic activity of neuronal cells. This is why mitochondria are studied as a key link in the mechanisms of cell death in ischemic stroke, and their importance as a therapeutic target has already been emphasized [69]. An increase in damaged mitochondria, disruption of their membranes, and their physical destruction promote the activation of reactive oxygen species (ROS)-dependent inflammatory cascades, for example, via release of nucleotide-binding oligomerization domain-like receptors (NLR), leucine-rich repeat (LRR) proteins, and pyrin domain-containing protein 3 (NLRP3) [52,70]. After ischemic injury and reperfusion period, disturbances in mitochondrial membrane potential, increased production of ROS, disruption of calcium homeostasis, and release of Cytochrome c (cyt c) lead to initiation of apoptotic pathways of cell death [56]. These processes underlie early neurodegeneration, i.e., loss of functionally active neurons, reduction in the firing and responsiveness of surviving neurons, and severe decrease in the synaptic dynamics [71].

On the other hand, the process of mitophagy is activated, which can prevent progressive neuronal damage by reducing the severity of oxidative stress and inflammatory response. Targeted modulation of mitophagy is a promising approach for protecting neurons in ischemic stroke. For example, drugs that inhibit the respiratory chain in mitochondria and modulate mitophagy, including metformin, have therapeutic effects, in particular in diabetic patients with post-stroke complications [72,73]. Some recent papers demonstrated the therapeutic effects of resveratrol, a naturally occurring polyphenol, in neuronal regeneration after ischemic stroke because of its anti-oxidative and anti-inflammatory properties [74]. However, other studies did not confirm that resveratrol can improve recovery from stroke in patients who suffered from prolonged ischemia episodes in the area of the middle cerebral artery, as evaluated by either the National Institutes of Health Stroke Scale (NIHSS), Modified Rankin Scale (MRS), or Barthel Index [75]. Therefore, translating mitophagy-associated findings into clinical practice requires in-depth studies of molecular pathways, pharmacodynamics, and well-designed human trials [76].

Restoration of mitochondrial function is ensured by the coordinated work of mitophagy, mitochondrial dynamics, and mitogenesis. Particular attention is paid to the regulatory pathways for mitophagy via the PTEN-induced kinase 1/E3 ubiquitin ligase Parkin (PINK1/Parkin) mechanism, which is involved in the quality control of mitochondria [77,78], and BNIP3L/NIX-mediated and FUNDC1-mediated molecular pathways, which serve as the receptor of mitophagy and are utilized in neuronal reparatory mechanisms [79,80,81]. Disruption of these processes contributes to a cascade of initiation of inflammation and cell death. Some pharmacological agents as well as traditional Chinese medicine can activate various neuroprotective signaling pathways, including AMPK/mTOR/ULK1 and LKB1-AMPK-SIRT1 [82,83]. It is equally important to achieve balanced mitophagy because an insufficient or excessive mitophagy can exacerbate neuronal damage. Therefore, the development of selective therapeutic agents that target mitochondrial processes and clinical trials with a personalized approach to stroke therapy are needed.

In addition, recovery from stroke is typically accompanied by some degree of endogenous repair. Endogenous stem cells, glial cells, microglia, and even endothelial cells and pericytes participate in the repair processes [63]. This is regulated by numerous pathways, including epigenetic modifications and upregulation/downregulation of microRNAs (miRNAs) and other non-coding RNAs [84,85]. For example, studies in knockout animals show that stroke is associated with significant changes in long non-coding RNA (lncRNA) profiles and lncRNA-mRNA co-expression networks in neural stem cells [86]. In addition, specific miRNAs are responsible for regulating anti- and pro-inflammatory responses following mitochondrial damage after prolonged and extensive oxidative stress; thus, it is important to elucidate the mechanisms of their expression [85].

MSCs secrete a variety of molecular factors that participate directly or contribute to these mechanisms, so they can target pathologically altered pathways and either silence or revert them to prevent excessive damage and death of brain neurons. Because MSCs of different origins have different molecular signatures of secretome, their regenerative and reparative capacities differ as well. In the next two sections, we highlight molecular mechanisms, functional roles, and (pre)clinical applications of MSCs, with a separate discussion devoted to delving into the therapeutic potential of WJ-MSCs in treating post-stroke injury of brain neuronal tissue.

## 3. Stem Cell-Based Regenerative Strategies to Restore Neuronal Activity in Post-Stroke Patients

### 3.1. Stem Cells (Other Than WJ-MSCs) in Post-Stroke Therapeutic Applications

Studies earnestly show that stem cells and their derivatives (secretome, exosomes) have the potential to promote neuronal repair by providing neuroprotection and stimulating neurogenesis, angiogenesis, and immunomodulation [87,88,89,90]. The most studied and safest cells to date are mesenchymal stem cells (MSCs), particularly those derived from bone marrow (BM-MSCs), adipose tissue (AD-MSCs), and umbilical cord (UC-MSCs) [66,91,92,93,94,95]. Earlier in vitro studies with cultures of MSCs from different sources revealed that despite their generally indistinctive morphology, the UC-MSCs have the longest life-in-culture and perform the highest number of passages, while BM-MSCs are the inferior ones in this context [91]. This finding explicitly indicates which type of MSCs may be more suitable for the generation of larger amounts of cells for therapeutic purposes.

Experimental findings in animal models of stroke showed that BM-MSCs and AD-MSCs act through the mechanisms of regulation of Vascular Endothelial Growth Factor (VEGF), major synaptic vesicle protein p38 (synaptophysin), and glial fibrillary acid protein (GFAP), as well as through the recruitment of oligodendrocytes and promotion of neurofilament reconstruction [92]. The preconditioning of these MSCs can further elevate their levels of expression of neurotrophic factors and the factors that contribute to the neuronal tissue regeneration [96,97,98]. BM-MSCs are also sensitive to late-stage inflammatory signaling like High mobility group box 1 protein (HMGB1) and S100 Protein family, which stimulate migration of stem cells to the site of inflammation and facilitate their differentiation via p38 MAPK signaling pathway [99]. Subsequently, the accumulation of BM-MSCs at the site of inflammation reduces the expression of S100 proteins, preventing excessive stem cell migration [100]. Moreover, MSCs can directly transfer their functionally active mitochondria to the injured neuronal cells via tunneling nanotubes: this mechanism has been shown in vitro in rat and human BM-MSCs [101,102]. This mechanism constitutes the more complex cell-to-cell exchange by organelles, which is distinct from signaling pathways and may represent a more robust way, as the injured cells receive ready-to-use energetic machinery from the healthy MSCs [103,104]. Additionally, transplanted MSCs can establish direct contact with target cells via gap junctions, facilitating the transfer of small signaling and regulatory molecules—this mechanism may directly regulate inflammation suppression in the post-stroke brain tissue [105]. This mechanism of gap junction-mediated communication between cells is involved in reparative angiogenesis via cell–cell regulation of the secretion of proangiogenic factors [106]. Moreover, this MSC-induced regulation of gap junction communication implicates another very important mechanism of the control of neuronal tissue composition, because different types of cells affect both their physical connection to each other and the growth of certain cells like microglial cells [107]. Of interest, this cell-to-cell contact likely contributes to the modification of genes responsible for cell proliferation, growth, and death, which in turn determines the fate of the target cell [107].

Induced pluripotent stem cells (iPSCs) and embryonic stem cells (ESCs) are also being considered as a source for regenerative capacity, although their use is associated with ethical and immunoregulatory difficulties. At now, iPSCs are considered a promising strategy for treating various diseases and pathological states, including neurological disorders like Parkinson’s and Alzheimer’s disease [108,109]. Despite their limited use in clinical practice, preclinical experiments showed high regenerative potential of human ESCs primarily cultured to induce differentiation into neural progenitor cells and then intravenously infused into rats 7 days after the stroke [110]. The treatment by these cells affects neuroinflammation pathways—NF-κB, Janus kinase/signal transducer and activator of transcription (JAK/STAT), NLRP3-mediated inflammation, mitogen-activated protein kinase (MAPK) signaling—and initiates faster recovery by over-expression of various proteins and signaling factors; in addition, the mechanism of extracellular vesicles to exchange the factors in paracrine regulation is utilized [111,112,113]. Systemic (intravenous) injection of small ESC-derived extracellular vesicles in transient cerebral ischemia reduced the levels of inflammatory cytokines and leukocyte infiltration to the infarcted brain area, and therefore prevents sustained and prolonged neurological damage [111]. However, challenges exist regarding the use of allogeneic vs. autologous iPSCs, as the cells may compromise immune reactivity after infusion [108].

Another type of MSCs—the so-called multilineage differentiating stress-enduring (Muse) cells, which reside in connective tissue and peripheral blood—represent the primary source for iPSCs and reside in mesenchymal tissues; owing to this feature, these cells may differentiate into the cells of all germ layers [114,115]. The cells also possess high stress-resilience, may survive in prolonged periods of hypoxic conditions, and generate iPSCs of fibroblast populations, which is especially important for regenerative treatment [114]. These unique properties make them a good source for regenerative medicine in patients with a variety of pathologies, including stroke and myocardial infarction [11,115,116].

Furthermore, different types of MSCs can express factors closely related to the recovery and proliferation of neuronal cells. For example, Csf1—the factor associated with the proliferation of microglia—is expressed differently even in the subpopulations of AD-MSCs [117]. Therefore, within the same set of MSCs, one subpopulation may be more potent in brain tissue recovery compared to another subpopulation. Similarly, if MSCs express a higher amount of C-C motif chemokine ligand 2 (CCL2), their administration results in better recovery of damaged brain tissue because of higher amounts of CCR2 in the damaged areas—this effect of homing capacity of MSCs is ultimately important in cell-based therapy [118].

It should be noted that the outcomes of stem cell therapy may depend on the type of administration. Preclinical studies in small animals showed high efficacy of intranasal administration of stem cells due to enhanced penetration of the blood–brain barrier (BBB) by active substances secreted by the cells [98,119,120]. In contrast, intravenous administration of MSCs is most often used in human trials, especially in the acute and subacute phases of stroke, while intracerebral and intra-arterial methods are used in the chronic period for more precise targeting of affected areas of the brain [92,121,122,123,124,125]. A recent study found that intravenous infusion of umbilical cord MSCs resulted in better outcomes in both NIHSS and Functional Independence Measure (FIM) than those of intrathecal injection [126]. On the other hand, another systematic review evaluated 21 available clinical sources for MSC treatment and summarized that intracranial delivery of the MSCs provides better post-stroke recovery, but this way was also most dangerous in terms of neural complications due to its higher invasiveness [127]. A more recent meta-analysis (evaluated 17 clinical studies in stroke patients) revealed that the intravenous route of administration was as safe as other non-systemic routes, while its long-term outcomes were inferior to these methods [128]. Of note, the outcomes in intracranial administration can be additionally modulated by a pretreatment of the damaged brain area. For example, preclinical studies show that the effect of direct MSCs infusion into the damaged brain area is amplified under preceding cooling of that area [121].

Additionally, whatever is, the way of administration of live stem cells, their highly limited ability to cross the BBB, and rapid in vivo clearance in the spleen, lungs, and other organs represent a substantial clinical challenge [124,129]. This feature of live cells is highly distinctive from the ability of cell-free material to cross biological barriers much more easily and to avoid substantial systemic clearance (see next subsection). Compared to the acellular substances, transplanted live stem cells can migrate through the BBB only in a limited amount that is driven by their homing ability and transient attachment to the vessel walls; to a great extent, the attached cells are returned to the systemic circulatory system [130]. Additionally, excessive accumulation of stem cells in non-target organs may induce adverse remodeling, e.g., in pretumor conditions where stem cells accumulate better in the tumor microenvironment and may accordingly undergo excessive proliferation and promote tumor development there [131].

### 3.2. Stem Cells (Other Than WJ-MSCs) Secretome and Exosomes

It is widely accepted now that the regenerative effect of stem cells is achieved primarily through paracrine effects (release of growth factors, exosomes, or even functionally autonomous organelles) rather than direct transdifferentiation into neurons [102,103]. Indeed, MSC-derived extracellular vesicles (EVs) represent one of the most efficient ways of cell–cell communication due to their completely natural production. Their formation, loading, intracellular transportation, and release are strictly regulated by intracellular machinery [132]. They can carry a broad spectrum of biologically active components, including proteins, diverse RNA species and their fragments, nucleic acids, metabolites, growth factors, and lipids synthesized inside the parent cell, thus mediating paracrine effects of parental MSCs [133]. This heterogeneous cargo underlies the augmented functional versatility of EVs as they can carry not only signal factors and regulatory molecules but also ready-to-use proteins like receptors and ion channels, which easily integrate into the membrane of the recipient cell.

Current classification divides EVs into three main subtypes—microvesicles, exosomes, and apoptotic bodies—which differ in their origin, release pathways, morphology, cargo composition, and functional features. Apoptotic bodies are less involved (if any) in regulatory regenerative pathways. In contrast, microvesicles and exosomes represent a pool of highly mobile carriers, but their actual functional capacities differ, in particular in the context of post-stroke effects (Table 1). While microvesicles are involved in tissue repair, exosomes represent more diverse functions, including precise control of angiogenesis, neurogenesis, and fine-tuning of inflammation resolution at the site of injury [133]. One of the distinct features of exosomes vs. microvesicles is that the former can easily cross the BBB due to their smaller dimensions [134]. Because almost any living cell undergoes the release of its own EVs and is permanently targeted by EVs secreted by other cells, it is important to consider how different cell types may regulate each other. The typical scenario is the exosome-based (inter)action of MSCs and MSC-targeted host cells. The extent of this communication depends on the inherent secretory potential of cells, including the capacity for EV production. For example, EVs generated by terminally differentiated cells tend to carry limited regenerative potential, whereas vesicles secreted by MSCs reproduce many of the therapeutic functions attributed to cells themselves [135]. Furthermore, exosomes secreted by stem cells typically carry higher concentrations and diversity of growth and trophic factors, as well as exhibit a higher propensity to cross the BBB compared to the exosomes released by other “adult” cell types [136].

Exosomes are the most mobile and biologically active subtype of EVs. They are present abundantly in the blood and can also be detected in high amounts in other biological fluids like urine, saliva, breast milk, and cerebrospinal fluid. The molecular profile of exosomes includes numerous signaling factors with selective tropism toward particular cell types [137]. The presence of diverse exosomal populations in extracellular fluids allows fine modulation of different recipient cells simultaneously [138]. When cells experience stress or damage, they release exosomes enriched with pathological molecular signatures, including proteins and microRNAs characteristic of disease states. This explains their pivotal contribution to tumor progression, metastasis, and also to numerous non-cancerous disorders, as well as their unique role as a precise and personalized prognostic biomarker [139,140,141,142]. Cell secretome, in turn, consists of numerous types of soluble factors, including growth factors, cytokines, chemokines, interleukins, prostaglandins, angiogenic mediators, hormones, etc. Despite the fact that these are generally smaller molecules compared to those carried by exosomes (like complete membrane-spanned proteins), the secretome is a vital mate for exosomal signaling [87,88,143,144,145].

Importantly, secretome/EVs may act both locally within a limited space and distantly across various organs, being disseminated via the circulatory system (predominantly exosomes vs. microvesicles due to their size inequality). In this way, EVs contribute to tissue homeostasis and support normal regulatory pathways under a healthy state [132,134]. In pathological conditions like acute release of DAMPs by injured neuronal cells or a persisting inflammatory environment, the role of EVs may be specifically contributing [132,143]. Indeed, MSC-derived exosomes regulate the activity of resident immune cells in the affected areas of the brain by secretion of immunomodulating factors, which is considered a key mechanism for exosomal action [146]. Preclinical studies that have been made over the last decade show that MSC-derived exosomes are a safe and effective treatment like MSCs themselves, as well as they represent tissue-specificity, which may facilitate their therapeutic usage [147,148,149,150].

In the previous subsection, we noted a challenge that certainly limits the use of live stem cells as a therapeutic product—their high clearance in organs and tissues. In contrast to live stem cells, MSC-derived EVs are cleared to a much lower extent. Notably, EVs show dynamic distribution in the organs rich with macrophages while exhibiting low accumulations in the spleen, lungs, and liver compared to the intact stem cells [151]. Furthermore, it has been shown in a mouse model of ischemic stroke that MSC-derived EVs tend to accumulate more specifically in infarcted vs. non-infarcted areas of the brain [152]. In addition, EVs do not cause local cytotoxicity but inhibit excessive fibrosis in the damaged tissue, preventing scar formation [153,154]. However, some animal studies showed that their accumulation and clearance are affected by the number of subsequent administrations contrarily to live stem cells—repeated intravenous infusion of EVs may induce their elevated clearance [155].

### 3.3. Preclinical and Clinical Application of MSCs (Other Than WJ-MSCs) and Their Secretome/Exosomes

Despite many reports and clinical trials being available for using MSC-based therapy in post-stroke rehabilitation in the last two decades (both preclinical and clinical studies), there is some extent of controversy in the results, especially regarding long-term effects. The controversies may stem from using various types of MSCs: BM-MSCs, UC-MSCs, and AD-MSCs; other types, such as hematopoietic stem cells and mixed populations of bone marrow mononuclear cells (BM-MNCs), are also used. Therefore, the efficacy and safety of various types of stem cells, different routes of administration, and post-stroke intervals of administration must be carefully analyzed among the available clinical trials. Also, different types of neuronal damage and neuropathological states may require different types of MSCs [156,157,158]. For example, human amniotic epithelial stem cells express superior immunotolerance and better antioxidant activity vs. WJ-MSCs; therefore they application in neuroinflammatory conditions may be beneficial [156]. Accordingly, BM-MSCs and AD-MSCs with their remarkable regenerative capacity revealed high efficiency in functional recovery of damaged neuronal tissue in spinal cord injury and brain injury [157,158].

Preclinical studies demonstrate the positive effect of MSCs on the recovery of motor and cognitive functions in animals with either cerebral artery occlusion-induced ischemic stroke or traumatic brain injury [66,93,159,160,161]. Transplantation of genetically modified MSCs overexpressing IL-10 in a model of traumatic brain injury was found to reduce neuroinflammatory markers—e.g., shift from classical inflammation with expression of CD86 to alternative inflammation with more prevalent expression of CD163—and enhance mitophagy and autophagy [160]. The use of BM-MSCs, preactivated by interferon gamma (IFN-γ), in a stroke model in rats significantly reduced the damaged brain area and prevented excessive activation of microglia via recruiting oligodendrocyte progenitor cells and their induction to the myelin-producing cells [66]. In another study, BM-MSCs were transfected with miR-21a-5p, and the reparative potential of BM-MSC-derived exosomes was evaluated [162]. It was found that exosomes produced by BM-MSCs with overexpressed miR-21a-5p had better reparative effects because of a direct inhibitory effect on the Toll-Like Receptor 2 (TLR2)/NF-κB pathway involved in the inflammation in brain tissue. A similar finding was obtained in a mouse model of ischemic stroke, where treatment by miR-671-5p targeting the NF-κB/MMP-9 (matrix metalloprotease 9) pathway alleviated adverse structural alterations in neuronal cells associated with their degradation and recovered brain tissue more efficiently [163]. The positive effect of MSCs was confirmed in the animal models of comorbid stroke, e.g., in rats with nicotinamide- and streptozotocin-induced hyperglycemia followed by permanent occlusion of the middle cerebral artery [93]. Using labeled BM-MSCs injected into the affected brain area of ischemic stroke rats, it has been confirmed that the cells actively migrate to the damaged neuronal cells and correlate with better recovery of the rats [164]. Importantly, BM-MSCs that resided in the injured areas of the brain did exhibit neuronal phenotype, e.g., they started expressing neuron-like firing activity and neuronal marker NeuN.

Despite the general improvement in post-stroke recovery in animal models, clinical studies and trials often report minor or no effect of BM-MSC or AD-MSC therapies. For example, the intravenous infusion of autologous BM-MSCs twice in a 4-week interval to patients with chronic stroke complications was found safe and resulted in slightly better recovery within one year of observation compared to the placebo group [165]. In patients with chronic conditions (while the stroke occurred 0.6 to 24.8 years before the treatment for the total cohort of patients, *n* = 36), the infusion of allogeneic ischemia-tolerant MSCs also resulted in improved recovery according to NIHSS and Barthel Index [166]. In this study, the safety of cell infusion was uncompromised with up to 1.5 × 10^6^ cells per kilogram body weight. In patients with sub-acute and sub-chronic post-stroke state, i.e., up to 8 months after the stroke, the treatment with UC-MSCs improved both NIHSS and Rivermead Assessment Scale significantly over the 12-month period of follow-up [167]. On the other hand, a small-sized phase II clinical trial with four patients treated by AD-MSCs within 2 weeks after stroke (i.e., early intervention) did not reveal any positive effect vs. the placebo group; moreover, a non-significantly lower median NIHSS score was found in the treated patients [168]. Similarly, intravenous infusion of preconditioned autologous MSCs in patients with sub-acute stroke did not produce improvement of the MRS score 3 months after the infusion [122]. Larger studies also show contradictory results. A phase II/III study enrolled over 200 acute ischemic stroke patients to evaluate the safety and outcomes of early treatment by allogeneic multipotent adult progenitor cells, i.e., in 18–36 h of the stroke onset [169]. The finding was no difference between MSC-treated and non-treated patients concerning any measure used. The medium-term infusion, 3-to-10 days post-stroke, of umbilical cord blood, presumably containing a high amount of MSCs, also did not gain any additional benefit to the patients if evaluated by NIHSS, MRS, or Barthel Index [170]. Similarly, it has been shown that intravenous administration of preconditioned autologous MSCs provides a cell–substrate for protection of the corticospinal tract and better recovery in the (sub)ischemic stroke patients (enrolled either within 2 weeks or 90 days post-stroke) as evaluated by Fugl-Meyer assessment score for motor function or NIHSS [122,171,172]. However, in these studies, the key limitations included small sample sizes and incomplete blinding.

There are several clinical trials (phase I/II), both ongoing and completed, in which the cell-based therapy is used to treat patients with ischemic/non-ischemic stroke [11]. In many of them, the source for MSCs is the bone marrow, while neuronal stem cells are also used. The main evaluation aims are safety and tolerability of the intervention, i.e., short-/medium-term effects rather than long-term effects. According to several meta-analyses, available large clinical trials demonstrate the safety of the MSC-based therapy and report moderate positive effects on neurological deficit scales (NIHSS, MRS, Barthel Index) [95,173,174,175,176]. On the other hand, the positive effect of infusion of BM-MNCs, which are a heterogeneous population of stem and progenitor cells, was distinct from untreated patients 3 months post-infusion, while no difference was reported 6 months after the treatment [175]. A matched-control clinical trial, in which 50 patients with traumatic brain injury were administered intrathecal BM-MNCs infusions, showed better restoration of motor and cognitive scores, with a minimal rate of adverse effects [177]. The therapy by BM-MSCs significantly improved neurological function in patients after ischemic stroke, as compared to the untreated patients, but in various scales (NIHSS, Fugl-Meyer assessment, Functional Independence Measure), the significance was found at different time points post-treatment—this indicated a strong influence of the selected assessment method on the measured outcome [175]. Another meta-analysis of outcomes by 13 clinical trials in patients with acute and sub-acute stroke revealed better 3-month and 1-year MRS scores in MSC-treated compared to conservatively treated patients; however, the rate of adverse effects and mortality was unaffected by MSC treatment [95]. Similarly, a meta-analysis based on the data accumulated from thirty randomized and non-randomized clinical trials (approximately 600 patients in the treatment or control groups) concluded with better neurological outcomes and lower mortality in MSC-treated patients by both NIHSS and MRS scales [173]. Adverse effects, such as fever, were generally observed at low rates among the trials, and no severe adverse effects were reported. However, other studies report less prominent outcomes of the stroke treatment by MSCs. For example, a recent meta-analysis based on 18 clinical trials showed better outcomes in the treated vs. untreated groups only for MRS and Barthel Index, not for NIHSS [176].

Importantly, dosages, MSC types, administration routes, and outcomes evaluation criteria highly vary between the studies [178]. In addition, in general, small sample sizes result in a high variability in the reported therapeutic efficacy of MSC. All of these limit the development of a unified assessment of MSC efficacy and their wider clinical use, and require large multicenter randomized trials with strict standardization for cell type, dose, timing, and route of administration. Furthermore, the application of MSCs should follow its “therapeutic window”. The concept was proposed more than a decade ago, and it introduces an important factor specifically for MSC therapies—the current developmental stage of MSCs at the time of infusion or injection into a patient [179]. If the cells are used beyond the window, their efficacy may be reduced or even unpredictable. This important factor must always be taken into account when the effectiveness of the treatment is cross-compared between trials.

Similar to other therapies, cell-based therapy may be applied as soon as weeks post-stroke. However, the stroke-related consequences for motor activity, cognitive functions, and other principal features of the affected individual should be assessed prior to the treatments. This is ultimately important because stroke severity may result in different lesions in the brain and/or different brain areas affected by the shortage of oxygen supply [180,181,182]. Also, the time window between the stroke and the treatment is important for consideration because of the cumulative effect of post-stroke pathological remodeling, if the treatment was initiated in the late part of the subacute phase [29,183]. Furthermore, ischemic and non-ischemic stroke-specific inflammation markers—systemic inflammatory response index, neutrophil-to-high-density lipoprotein ratio, neutrophil-to-lymphocyte ratio, inflammation-related factors, signaling molecules, and enzymes, etc.—can be used to assess the level of post-stroke systemic inflammation in order to envisage or anticipate possible complications related to the treatment by MSCs [67,184]. Finally, currently implemented experimental strategies for treating stroke-injured brain tissue include co-cultivation of MSCs or their exosomes with various plant extracts from medicinal or traditional plants and berries, including traditional Chinese plant medicines [185,186,187].

## 4. Wharton’s Jelly Mesenchymal Stem Cells (WJ-MSCs) in Post-Stroke Therapy

### 4.1. Brief Overview of the Roperties of WJ-MSCs

**Figure 2:**
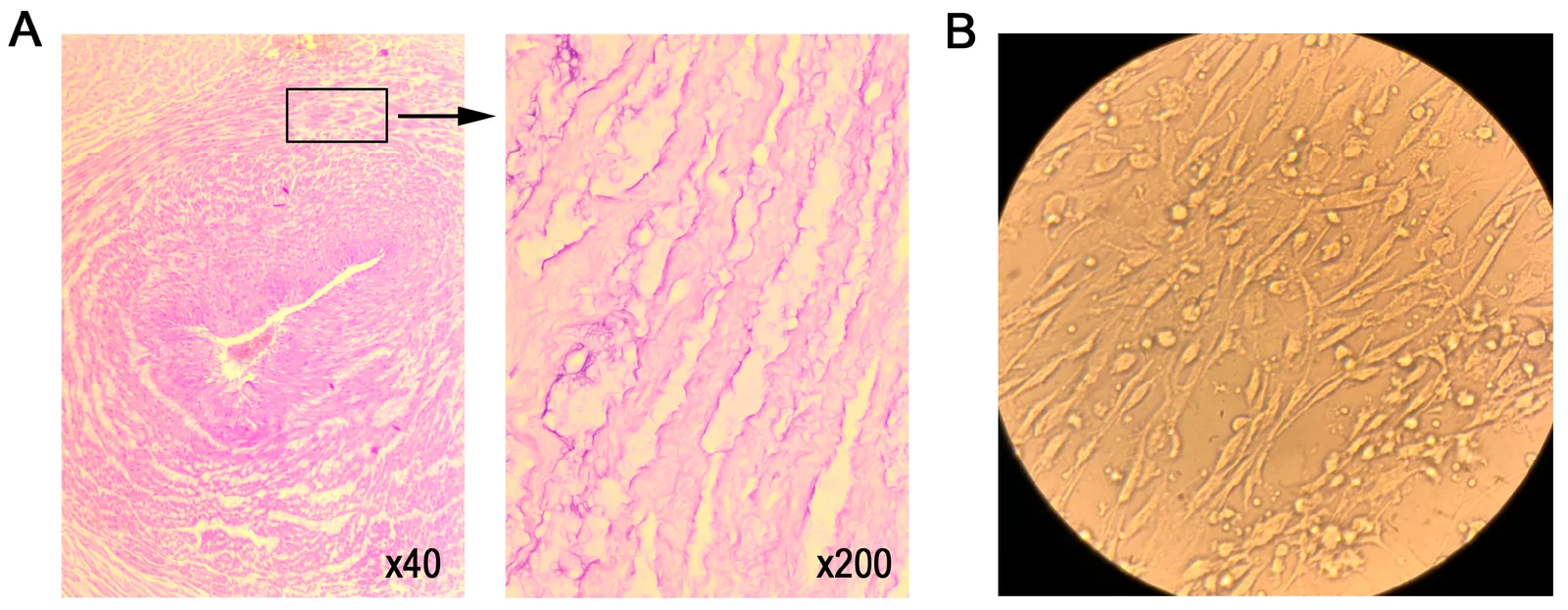
Histological structure of umbilical cord cross-section showing the lumen of vessel ((**A**) **left image**) and Wharton’s jelly around it ((**A**) **right image**, shows magnified area covered by the box in (**A**) **left image**), and typical appearance of Wharton’s jelly MSCs cultured in vitro (**B**). The image in (**B**) is adapted from [190] with permission.

The function of Wharton’s jelly is to protect the surrounding vessels of the umbilical cord from compression, twisting, and bending and therefore to maintain blood flow between the fetal and maternal circulations (Figure 2A). The mucosal connective tissue contains specialized fibroblast-like cells and some mast cells. The Wharton’s jelly is the main source of UC-MSCs (Figure 2B), with absolute amounts of ~5.5 million MSCs per one centimeter of umbilical cord or even higher [94,188,189].

A decade ago, it was proposed that UC-MSCs, in particular Wharton’s jelly derived mesenchymal stromal cells (WJ-MSCs), are the new gold standard in cell therapy [191]. Studies have shown that the gross morphology, proliferative capacity, and pluripotency expression profile of WJ-MSCs remain similar among all parts of Wharton’s jelly [189], whereas MSCs obtained from the umbilical cord membrane lost their proliferation earlier than WJ-MSCs [94]. More recently, it has been found that MSCs derived from different parts of the umbilical cord (including Wharton’s jelly) do display heterogeneous secretion of vascular markers, thus indicating that various parts of the umbilical cord should be selected for specific treatments [192]. This evidence correlates with findings that the proportion of MSCs with distinctive morphological features changes between different parts of the umbilical cord [193]. In addition, some data show that the cells isolated from the cord-placenta junction, another region of the umbilical cord, express the highest amounts of various pluripotency markers, even compared to WJ-derived cells [194]. Of note, if a freshly obtained Wharton’s jelly sample is frozen, it retains high capacity for generating large amounts of MSCs after thawing, in contrast to the capacity of a whole umbilical cord sample, which contains mixed cell populations [195].

Like other types of MSCs, WJ-MSCs exhibit classical characteristics of stem cells, such as pluripotency and self-renewal, but their stemness is considered to be most primitive, giving them the highest pluripotent potential compared to most other regions (except the cord-placenta junction) [194,196]. They represent high adherence in culture and express specific surface antigens (e.g., CD24, CD105, CD108, CD73, CD90), and they are able to differentiate into osteoblasts, adipocytes, and chondroblasts [94,188,194,197,198]. They do not express fibroblast-specific surface markers [188]. Thus, it discriminates WJ-MSCs from other cells in a heterogeneous population and lets obtaining cell fractions with little contamination by other cell types. Furthermore, WJ-MSCs exhibit low immunogenicity and high biological activity [156].

Compared to AD-MSCs or BM-MSCs, WJ-MSCs are known to have higher proliferative potential, lower oncogenic properties, and a more “primitive state”, i.e., represent themselves as more young cells with higher stemness [199,200,201,202,203]. For example, their doubling time is higher compared to BM-MSCs [194]. They intrinsically have higher expression of genes related to neurotrophics and neuronal maturation, making them a better candidate in neuroreparative therapy than BM-MSCs, which show a lower effect on neuronal growth [204]. Due to their embryonic origin, WJ-MSCs exhibit high expression of pluripotent markers (NANOG, Oct3/4, Sox2), suggesting greater differentiation and proliferation potential, and also diminished susceptibility to environmental factors and mutagens [205]. When MSCs isolated from bone marrow, adipose tissue, and Wharton’s jelly were compared after culturing in fetal bovine serum-supplemented media, the WJ-MSCs were superior in cell proliferation, and the BM-MSCs were inferior [199]. If seeded and cultured in a 3D scaffold (alginate/hyaluronic acid hydrogel) during ~30 days, the WJ-MSCs display better chondrogenic potential than BM-MSCs in the same conditions; also, the two cell types show different phenotypic profiles [206]. It has been proposed that differential expression of genes responsible for stimulation of cell proliferation and regulation of cell cycle (in particular Wnt/β-catenin signaling) in WJ-MSCs vs. BM-MSCs is associated with greater proliferative capacity of WJ-MSCs [200]. Also, compared to the BM-MSCs and AD-MSCs, WJ-MSCs produce higher amounts of chemokines, pro-inflammatory proteins, and growth factors that make them diverse in immune modulation pathways [207]. Importantly, WJ-MSCs may facilitate an anti-inflammatory response. For example, in co-cultured mature myeloid dendritic cells with human T cells, the addition of even a low amount of WJ-MSCs (1:100 ratio) induced a significant elevation of anti-inflammatory Transforming growth factor-beta (TGF-β) [208]. Compared to BM-MSCs, WJ-MSCs display greater immune-suppressive properties, likely because of different kinetics of TNF-α and INF-γ [209]. Moreover, activation of the negative co-stimulatory molecule CTLA-4 in phytohematoagglutinin (PHA)-activated lymphocytes was observed only if they were co-cultured with WJ-MSCs, not with BM-MSCs [209]. The co-culturing with WJ-MSCs significantly suppressed macrophagal mRNA expression of proinflammatory factors IL-1β, IL-6, and TNF-α [156]. In vitro cell culture studies showed that incubation in INF-γ triggers upregulation of chemokine ligands (CXCL9, CXCL10, CXCL11) and adhesion proteins (VCAM1, ICAM1) in WJ-MSCs, in contrast to fetal BM-MSCs and AD-MSCs [210]. The immunomodulatory properties of WJ-MSCs make them “immunoprivileged” cells because they express lowered major histocompatibility complex (MHC) class I/II molecules, CD40, CD80, and CD86 co-stimulatory factors [107]. It is also important to note that other stem cells may maintain their stem phenotype longer if co-cultivated with WJ-MSCs under certain conditions [211]—this indicates a capacity of WJ-MSCs for both self- and trans-modulation of stemness. Finally, WJ-MSCs display enhanced expression of genes responsible for angiogenesis and neurogenesis compared to BM-MSCs. For example, being co-cultured in vitro with endothelial cells in a model of ischemic stroke (via deprivation of oxygen and glucose in the medium), WJ-MSCs exhibited a better effect in terms of microvasculature formation compared to BM-MSCs [212].

As WJ-MSCs are extracted from a waste medical product—the umbilical cord—they represent a completely non-invasive and ethically acceptable source of stem cells. Compared to the MSC collection from tissues of adult donors, e.g., from bone marrow, the amount of initially extracted WJ-MSCs plus their higher proliferative potential warrants getting a sufficient amount of cellular material for a single treatment or a series of infusions [213,214]. Therefore, the use of Wharton’s jelly as a source of MSCs for culture and therapeutic purposes can be considered a more effective approach in terms of initial cell amount, cell renewal, and homogeneity/stability of cell properties, compared to other types of MSCs.

### 4.2. WJ-MSC-Derived Secretome and Exosomes

**Figure 3:**
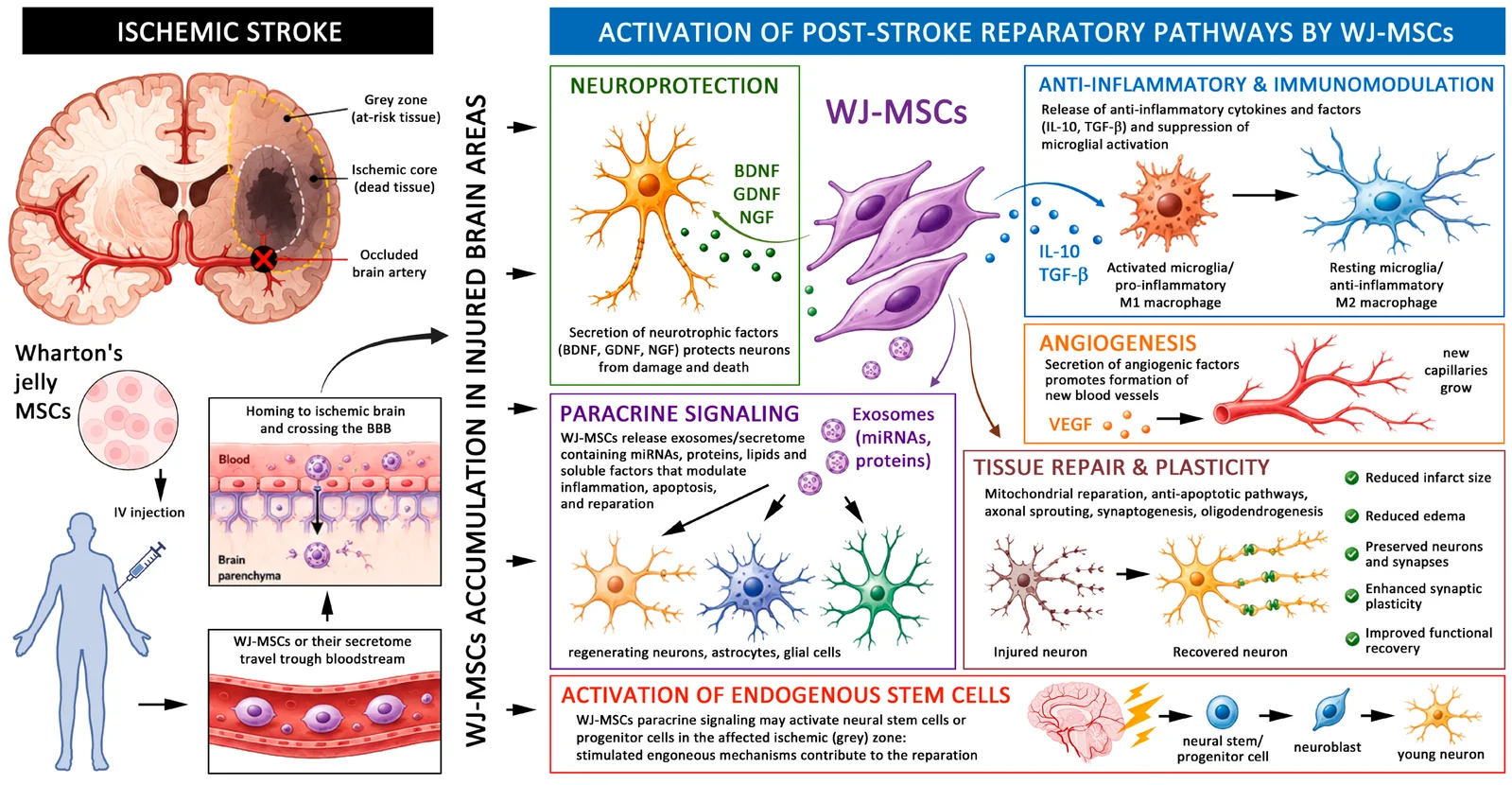
The principal pathways by which Wharton’s jelly mesenchymal stem cells (WJ-MSCs) can affect the target neuronal cells are the secretome, direct stem cell migration, and exosomes. The pathways represent a pool of mechanisms—based on the release of cytokines and chemokines, growth, neurotrophic, angiogenic, metabolic, and anti-apoptotic factors—collectively targeting restoration of impaired neurons, facilitation of their renewal, reduction in local inflammation, promotion of recovery of neuronal metabolism, attenuation of apoptotic events, and providing antioxidant protection. Through multi-targeted paracrine actions, WJ-MSCs create a neuroprotective, anti-inflammatory and pro-regenerative tissue microenvironment leading to preservation of survived brain tissue and improved functional outcomes after ischemic stroke. The following abbreviations are used in the figure: BBB—Blood–Brain Barrier; BDNF—Brain-Derived Neurotrophic Factor; GDNF—Glial Cell Line-Derived Neurotrophic Factor; IL—Interleukin, MSC—Mesenchymal Stem Cell; NGF—Nerve Growth Factor; TNF—Tumor Necrosis Factor; VEGF—Vascular Endothelial Growth Factor.

It has now been proven that the primary therapeutic effect of WJ-MSCs, like other MSCs, is mediated by their secretome and exosomes [102,201], i.e., via secreting of soluble factors like cytokines, chemokines, growth factors, and extracellular vesicles, e.g., exosomes with proteins and microRNAs (Figure 3). Compared to other MSCs, WJ-MSC secretome/EVs demonstrated the most promising characteristics for therapeutic application, particularly in the context of inflammatory and degenerative diseases [215]. The WJ-MSC-derived secretome contains cytokines, chemokines, and growth factors such as granulocyte-macrophage colony-stimulating factor (GM-CSF), IL-1α, IL-6, IL-8, IL-2, IL-7, IL-12, IL-15, MCP-1, MIP-1β, and PDGF-AA, which are involved in proliferation, differentiation, tissue remodeling, and immune cell mobilization [216]. Accordingly, WJ-MSC-derived EVs contain a wide range of proteins involved in regeneration, angiogenesis, immune response modulation, and apoptosis suppression, including alpha-2-macroglobulin, serum albumin, and heat shock proteins [215,217,218,219].

The neuroprotective effect of the WJ-MSC secretome (as well as the secretome of BM-MSCs) is characterized by the ability to protect neurons and resident macrophages from glutamate-induced toxicity and enhance their viability [220,221,222,223]. Furthermore, secretome components, including Brain-Derived Neurotrophic Factor (BDNF), Tumor Necrosis Factor (TNF), fibroblast growth factor 2 (FGF-2), insulin-like growth factor (IGF), glial cell line-derived neurotrophic factor (GDNF), Vascular Endothelial Growth Factor (VEGF), and Nerve Growth Factor (NGF), activate mechanisms of neurogenesis, angiogenesis, and remyelination, and also promote the restoration of neuronal connections and the reduction in neuroinflammation [87,224]. It has been shown that WJ-MSC secretome contains high levels of various growth factors; especially important that the content remains high in freeze-dried secretome embedded into carbomer gel matrix [225]. Furthermore, secretome components contribute to the restoration of energy metabolism and the preservation of neurons from death by reducing mitochondrial dysfunction and regulating mitophagy processes (via the PINK1/Parkin and BNIP3/FUNDC1 pathways) [77,78,79,80,81]. The antiapoptotic and antioxidant activity of the secretome plays an important role in suppressing ischemia–reperfusion damage. Intravenous administration of WJ-MSC exosomes to an experimental model of ischemic stroke in rats has been shown to protect cortical neuronal cultures from glutamate-induced toxicity and to increase levels of anti-apoptotic proteins, such as Bcl-2 and Bcl-xL, reducing the number of TUNEL-positive (dead) neurons in brain tissue [226]. Lyophilized secretome gel reduces levels of hydrogen peroxide and nitric oxide, exhibiting high antioxidant activity that protects cells from ROS-mediated oxidative stress [225]. Secretome contains angiogenic mediators, such as VEGF and platelet-derived growth factor-AA (PDGF-AA), which affect the vascular endothelium and improve vascular remodeling [216]. Compared to AD-MSCs, the secretome of MSCs obtained from umbilical cord (which includes WJ-MSCs) was superior in its effects on cell density and metabolic viability in primary cultures of hippocampal neurons exposed to the conditioned media [227]. In terms of its effect on glial activation and inflammation, the WJ-MSC-derived secretome has strong immunomodulatory properties. After ischemia, DAMPs secreted by damaged cells in neuronal tissue activate microglia and exacerbate neuroinflammation [59,61]. Secretome of WJ-MSCs regulates the release of proinflammatory interleukins and inhibits inflammatory cascades, such as the NLRP3 inflammasome [52,69]. The WJ-MSC secretome exhibits pronounced immunomodulatory, anti-apoptotic, angiogenic, and anti-inflammatory properties. It can modulate the cellular microenvironment, promote tissue regeneration, and suppress pathological inflammation.

Exosomes represent a more complex physico-chemical structure compared to the secretome due to the presence of specific membrane-bound proteins (endosomal proteins, tetraspanin protein family, etc.) [218]. Moreover, they not only contain various miRNAs but are also found to be performing active sorting of the exosomal miRNA in a manner that makes the exosomal miRNA profile different from that of the parent cell [228]. It is important for modulating the expression activity of the target cells. Using a miRNA-mediated mechanism, WJ-MSC exosomes promote expression of anabolic markers such as aggrecan and type II collagen, while simultaneously reducing the expression of the proinflammatory cytokine IL-6 and matrix metalloproteases and other specific enzymes that degrade the extracellular matrix—MMP-1, MMP-13, ADAMTS-5 and others [229]. This demonstrates the ability of these vesicles not only to restore cells in the extracellular matrix but also to prevent neuronal cell degeneration, maintaining their phenotype and functional activity. WJ-MSC exosomes possess significant therapeutic potential due to their microRNA and cytokine content, which regulate inflammation and regeneration and prevent neurodegradation in the brain white matter [229,230]. Exosomes isolated from WJ-MSCs contain high concentrations of proteins associated with the activation of complement and coagulation pathways, including fibrinogen and heat shock proteins [202,231]. These proteins play a key role in regulating inflammation and wound healing. Furthermore, exosomes from WJ-MSCs can increase levels of the neurotrophic factors bone morphogenetic protein 7 (BMP7) and GDNF in the brain and stimulate endogenous stem cell proliferation, neuronal differentiation, and synaptogenesis via molecules such as BDNF and NGF [226,232]. In addition, the cells secrete anti-apoptotic factors (Bcl2 and Bcl-xL), which further ameliorate the damage of survived brain cells [226]. Functional studies in vitro have shown that WJ-MSC exosomes are significantly more effective in stimulating the activity of fibroblasts and promoting the synthesis of collagen [233]. This demonstrates the high regenerative potential of WJ-MSC exosomes, making them a promising tool for clinical applications in various aspects of regenerative medicine [202].

A unique property of exosomes is the carrying of microRNAs and proteins to damaged brain cells. These vesicles contain lipids, regulatory proteins (adhesion molecules, enzymes) within or on the surface, as well as miRNAs and DNA fragments, which makes them a promising therapeutic drug [87,202,228,234]. It was demonstrated that WJ-MSC-derived exosomes prevent excessive liver fibrosis via the transfer of miRNA-133a, which actively regulates fibrin production, to the target cells [235]. If MSC-derived exosomes are preconditioned by incubation in miR-133b-containing media, they can attenuate the proliferation and migration of glioma cells in vitro [236]. WJ-MSC-derived exosomes can protect primary hippocampal cells from oxidative stress by reducing their intracellular ROS level in the co-cultured condition [237]. It is noteworthy that the conditioning of WJ-MSCs in culture may affect the regulatory secretome profile of WJ-MSC-derived exosomes [98,238]. For example, hypoxia-preconditioning of WJ-MSCs amplified regenerative potential in the treatment of renal tissue [239] and in the treatment of stroke-induced brain injury in animal experiments, in particular by promoting cerebral angiogenesis [98,240]. Because transient or intermittent hypoxia displays a positive effect on microglial cells and their polarization to an anti-inflammatory phenotype [241], it has been proposed that exosomes produced by hypoxia-preconditioned MSCs may exhibit better immunomodulatory properties than those of the non-preconditioned cells [97]. Moreover, a recent preclinical study in rats after middle cerebral artery occlusion found that systemic infusion of exosomes, obtained from UC-MSCs initially pretreated in a medium containing infarcted brain tissue extracts, resulted in better endovascular remodeling and decreased apoptotic events [242]. Importantly, this was due to the elevated migration of the “pretreated” exosomes to the damaged tissue, compared to the migration rate of “untreated” ones. These findings open a way for modulating the exosomal activity according to the personalized needs of the patient [97,232]. On the other hand, WJ-MSC-derived exosomes may be a useful therapeutic agent for in vitro modulating phenotypic properties and stemness of other stem cells, like AD-MSCs, before their in vivo administration [211,243].

Some of the preclinical studies have confirmed the efficacy and high therapeutic potential of WJ-MSC-derived cell-free product in ischemic stroke animal models [228,244]. Administered exosomes penetrate the BBB, can be taken up by astrocytes, directly affect the levels of proinflammatory cytokines and apoptotic proteins, activate antioxidant mechanisms, and improve neurofunctional indicators [97,245]. Thus, the use of the secretome and exosomes of WJ-MSCs may represent a safe and highly effective therapeutic strategy with significant potential for the treatment of neurodegenerative and inflammatory diseases of the nervous system. On the other hand, according to a recent meta-analysis of clinical trials with application of MSCs, those obtained from Wharton’s jelly are rarely used, in addition to the overall low number of trials devoted to the MSC-based therapy of neurological diseases and disorders [128]. Further clinical studies are needed to expand knowledge about the potentially beneficial effects of WJ-MSC-derived acellular product.

It is also accepted that MSC-derived acellular product offers many advantages with just a few limitations, as compared to the cell-based therapies with their potential risks of immunological issues [246]. Two main limiting factors for MSC-derived products can be mentioned. First is the need for specialized and standardized protocols for collecting extracellular products (secretome, exosomes) from MSCs of various types. Second, in general, a greater number of infusions/injections of MSC-derived exosomes is required to attain an effect comparable with whole-cell MSC treatment [244]. Both factors increase the total costs for the cell-free treatment, especially the first factor, as it implies using specialized equipment for collection, filtration, separation, saturation, and storage of cell-free components.

### 4.3. Preclinical and Clinical Application of WJ-MSCs and Their Secretome/Exosomes

Numerous preclinical studies showed that WJ-MSCs represent a potent pool of cells in terms of the regulation of regeneration and healing in many organs and tissues. With specific focus on their reparative potential in the injured brain, studies indicate prominently beneficial effects of WJ-MSCs in experimental models of brain hypoxia-ischemia in neonatal or adult mice and rats [224,226,247,248,249,250,251,252]. Of note, their potential is remarkable even after intranasal administration in the animal models [247,248]. In a rat model of cerebral infarction, the transplantation of human Wharton’s jelly MSCs (WJ-MSCs) resulted in a smaller brain infarction and a limited activation of brain-specific immune cells (microglia), with better preservation of neurophysiological function in the animals [161,252]. Similarly, adenovirally transfected WJ-MSCs with elevated expression of miR-9-5p were found to both improve their chemotaxis to the infarcted area of the brain in a rat experimental model of ischemic stroke and be effective in the reduction in this area [253]. This effect is achieved via elevated secretion of NGF, BDNF, and IL-6 (anti-inflammatory and neuroreparative factors) and suppressing the secretion of IL-1 and IL-2 (pro-inflammatory factors) [253]. This effect was achieved as early as a few days after direct transplantation of WJ-MSCs into the ischemic brain of rats [252]. In another study, rats with cerebral artery occlusion to model ischemia–reperfusion damage in the brain were treated by WJ-MSCs prior to the occlusion [254]. Compared to non-treated ischemia–reperfusion rats, WJ-MSCs resulted in an enhanced expression of not only neurotrophic factors GDNF and BDNF but also Phosphatidylethanolamine Binding protein 1 (PEBP1), which is known to be involved in the regulation of kinase cascades like MAPK and NF-κB [254]. This WJ-MSC-mediated fine control of neurotrophic processes and inflammatory pathways in the affected zone facilitated the reduction in the infarcted area and resulted in improved memory and learning tests. Also, among the involved molecular mechanisms are the enhancement of CD31+/BrdU+ proliferating endothelial cells and the reduction in infiltration of macrophages into the infarcted zone of the brain [255]. It is important to note that the inherent plasticity of WJ-MSCs allows them to be preconditioned in vitro to adapt to the pathological environmental conditions in vivo. Thus, if preconditioned under hypoxic conditions in vitro, the cells acquire a secretory profile that is more beneficial to treat affected (damaged or injured) tissue where pathological hypoxia-associated remodeling is in action [98,224,256].

Clinical reports indicate the beneficial potential of using the secretome from umbilical cord-derived MSCs (including WJ-MSCs) in treating neurodegenerative diseases and neurotraumatic states [257], cardiac injury in myocardial infarction [162,258], lung diseases [259], liver fibrosis [260], facilitating deep wound healing in mediastinitis [190], etc. To date, WJ-MSCs and their secretome/exosomes have been used for various pathological states and conditions: for example, osteoarthritis [261,262], Alzheimer’s disease [257,263], autism spectrum disorder [264], amyotrophic lateral sclerosis [265], wound healing [202], regeneration of diabetic foot ulcers [225,266], and diabetes mellitus [267]. It has been shown that the efficacy of cell-based (using MSCs) and cell-free approaches (MSC-derived secretome/exosomal product) is essentially similar, and in some instances, the regenerative potential of the cell-free products is even higher [268]. For example, in a phase I clinical study on patients with traumatic brain injury, the application of WJ-MSCs intrathecally, intramuscularly, and intravenously resulted in the improvement of cognitive and motor function of the patients, with no adverse effects in a one-year follow-up period [269]. Recent clinical studies demonstrated the safety of WJ-MSC-derived exosomes in treating brain injury and spinal cord injury as well, with minor adverse effects and improvement of motor and cognitive functions [244,257,270].

While it is necessary to apply exosomes several times to acquire a therapeutic effect comparable to the cell-based infusions, acellular therapy by WJ-MSC-derived cell-free product offers several important advantages over traditional cell transplantation in clinical applications. First, this approach enables scalable production and quality control with precise dosages using technologies such as ultrafiltration and lyophilization [271]. Secondly, since the therapeutic effect is achieved through paracrine factors, the need for live cell transplantation is eliminated, which minimizes the risks associated with cell therapy, such as immune response (immunogenicity), vascular embolism, or tumor transformation (tumorigenic potential) [209,232]. Furthermore, the use of lyophilization technology significantly simplifies the logistics of product storage and transportation, making it a ready-to-use “off-the-shelf” product [225,272]. In addition, live cell-based therapies utilize various types of cells, with MSCs and freeze-dried plasma containing a variety of different cells as the most often used types of biomaterials [257]. Each cell type requires specialized protocols for isolation, culture, preconditioning, freezing, storage, thawing, and administration; this substantially complicates the process and substantiates high time- and resource costs. The production and administration of MSC secretome instead of live cells represents a good option to decrease all the costs.

Despite the positive results, current research has several limitations and gaps. The composition of the secretome and extracellular vesicles varies depending on the donor condition, the source of MSCs, and in vitro culture conditions, which affects the reproducibility of the results [273,274]. Heterogeneity of protocols (dose, time, and route of administration) makes it difficult to establish a uniform standard of treatment [178]. To ensure reproducibility and efficacy, issues such as lot-to-lot variability in secretome composition and the need for standardized protocols and quality control measures need to be considered. Clinical outcomes of applications of the MSC secretome/exosomes in various diseases are still under evaluation, especially for their long-term effects [274]. Finally, before wider introduction into clinical practice, it is necessary to standardize manufacturing processes and conduct multicenter clinical trials in accordance with Good Manufacturing Practice (GMP) standards.

In addition, various types of MSCs possess not only distinct features and functional capacities but also require different harvesting protocols, so their clinical application is still under investigation. Numerous in vitro, preclinical, and clinical studies allowed summarizing some key features and differences between the most used types of MSCs (BM-MSCs and AD-MSCs) and the relatively infrequent type in clinical practice, WJ-MSCs (Table 2). In regard to the post-stroke therapy, WJ-MSCs demonstrate highly beneficial features, including their highest proliferative capacity, prominent neurotrophic plus anti-inflammatory potential, and greatest extent of tolerogenecity. However, they are always allogeneic material vs. BM-MSCs and AD-MSCs, which are usually used in an autologous setting.

## 5. Prospects and Challenges of Implementation of WJ-MSC into Clinical Practice

Nowadays, the synergistic potential of cell-based and cell-free product therapy approaches with other types of neurorehabilitation is being grasped and implemented [275]. Particular attention is given to the concept of cell-free (exosomes, secretome) regenerative rehabilitation, combining this cell-free therapy with physical therapy, brain stimulation, and other restorative techniques [276]. However, as large clinical trials are still either ongoing or planned, it is necessary to carry out risk assessment and evaluate the therapeutic benefits of using MSC-derived exosomes in treating neurological disorders, in particular for post-stroke neurodegeneration [257].

Of note, most of the available clinical trials are focused on using BM-MSCs and AD-MSCs, either autologous or allogeneic, to treat neuropathologies. According to a recent meta-analysis of cell-based therapies for neurodamage (ischemic or traumatic), “adult” cells from these two sources prevail over other sources, with as little as 20% of clinical trials with UC-MSC/WJ-MSC for stroke and even much less for spinal cord injury [277]. Another meta-analysis of any-phase clinical trials focused on neurodegenerative diseases revealed only two trials with WJ-MSCs in the overall number of 50 analyzed trials [278]. The applicability of WJ-MSCs and their exosomal product remains to be widened in further studies to acquire more specific information on their efficacy in short- and long-term periods.

To successfully implement the secretome and exosomes of WJ-MSCs into clinical practice, strict standardization and certification of manufacturing processes, namely compliance with GMP and guidelines for the characterization of extracellular vesicles, are essential, as this ensures the product’s consistency and purity between batches [257,274]. Furthermore, determining optimal dosing regimens and methods of administration remains an important issue. In addition to intravenous administration, the development of non-invasive routes, such as intranasal, to improve BBB penetration could expand the therapeutic window [123]. From an ethical and regulatory perspective, Wharton’s jelly cells do not raise ethical concerns compared to embryonic cells, as they are derived from medical waste (umbilical cord). However, the legal framework for the regulation, registration, and certification of cell-free biological products needs to be established and optimized [213]. In the future, this therapy could be integrated into personalized medicine, where specific secretome cocktails tailored to the neurorehabilitation needs of each patient can be developed based on the detection of biomarkers combined with artificial intelligence data [232].

Next, one of the important stages in stem cell therapy is the preconditioning of MSCs before administration [97,239]. Injectable hydrogels, which also allow the encapsulation of stem cells or their secretome in an artificial “extracellular matrix”, are also used to facilitate local release and protection of MSCs from external influences, and these hydrogel-based vehicles are now considered a promising tool to accelerate regeneration [279,280,281,282]. The technology for producing a gel-like cell-free biomedical product based on the lyophilized secretome of BM-MSCs has recently been proposed [271]. Based on protein analysis and nanoparticle concentration, the MSC-derived secretome product formula with trehalose was selected as the most optimal for further functional experiments. Furthermore, the use of materials to imitate the extracellular matrix [282] and graphene scaffolds [283] provides support for cell growth and further in situ integration. The 3D bioprinting technology demonstrates the potential for studies in regenerative medicine, in particular for facilitating the re-growing of neuronal tissue in artificial 3D scaffolds [284]. Nanoparticle-based delivery of MSCs exosomes via non-systemic routes to avoid the BBB penetration problem is also considered a promising therapy [285]. With intravascular delivery, stem cells, the secretome, and/or nanoparticles capable of delivering bioactive molecules can be administered systemically. All of the presented technologies are aimed at restoring damaged areas of the brain and activating its own regenerative mechanisms. It should be noted that the transplantation of live MSCs or infusion of MSC-derived exosomes represents a better solution compared to treating with a mixture of certain cytokines and cell factors [10]. This is likely because the cells/exosomes are functionally more complex providers of numerous biologically active substances at a single time, with adjusted and orchestrated effects of each substance.

## 6. Conclusions

Rehabilitation therapy for stroke patients must take into account the interval from the onset of the stroke, damage to neurovascular tissue, focal loss of various tissue types, and the need to regenerate various functionally specialized cells across different brain segments. In this regard, the therapy with Wharton’s jelly mesenchymal stem cells and their secretome/exosomes is considered an innovative breakthrough in regenerative medicine. The lyophilized form of the cell content promotes the restoration of neurovascular communication, neoangiogenesis, vascular remodeling, and improves neuromotor function. However, large-scale clinical trials are needed to fully implement this technology in clinical practice and utilize its full potential. Future clinical research should be aimed at unifying GMP standards, determining optimal dosing regimens, and definitively demonstrating the long-term safety and efficacy of the WJ-MSC therapy in multicenter, randomized clinical trials. The combination of this promising cell-based approach with conventional physiotherapies, which can further be assisted or accompanied by digital technologies, may constitute an optimal rehabilitation strategy for stroke patients.

## Figures and Tables

**Table 1:** Principal distinct features of extracellular vesicles participating in intercellular communication.

Feature	Microvesicular Bodies	Exosomes
Dimensions	100–1000 nm (sometimes larger) Highly heterogeneous in size and shape	30–150 nm in diameter Relatively uniform in size and shape
Origin/mechanism of release	Biogenesis: Plasma membrane budding, driven by local changes in lipid and cytoskeletal organization	Biogenesis: Endosomal pathway, intraluminal vesicles released via exocytosis
Cargo	Proteins: Enriched in plasma membrane proteins, integrins, selectins, and cytoplasmic proteins Nucleic Acids: Rich in mRNA, miRNAs, and sometimes genomic/mitochondrial DNA Lipids: Enriched in phosphatidylserine (often flipped to the outer leaflet)	Proteins: Enriched in endosomal markers (tetraspanins) Nucleic Acids: Rich in specific small RNAs, miRNAs, and non-coding RNAs Lipids: Enriched in cholesterol, sphingomyelin, and ceramide
General functions	Local and rapid intercellular signaling Involved in blood coagulation, inflammation, and tissue remodeling Directly shed in response to cellular stress or activation	Long-range intercellular communication Involved in immune modulation, antigen presentation, waste disposal Excellent at crossing biological barriers (like BBB)
Level of action	Primarily act in the immediate microenvironment or local circulation near the site of cellular shedding	Highly stable; specialized for long-distance transport and targeting specific distant recipient cells via receptor-ligand interactions
Role in Post-Stroke Treatment	Tissue Repair: Mediate local tissue remodeling and angiogenesis Clotting Control: Can modulate local coagulation and endothelial cell activation post-ischemia *Note: Less frequently used than exosomes in targeted therapies due to delivery/homing challenges*	Neuroprotection: Readily cross the BBB to deliver neuroprotective miRNAs/proteins to ischemic zone Angiogenesis & Neurogenesis: Promote blood vessel growth and neural stem cell proliferation Anti-inflammation: Shift microglia from the pro-inflammatory M1 phenotype to the anti-inflammatory M2 phenotype

Note: BBB—blood–brain barrier.

**Table 2:** Comparative summary of “adult” MSCs (BM-MSCs and AD-MSCs) and “young” WJ-MSCs: principal features and (pre)clinical results and applications.

Wharton’s Jelly MSCs (WJ-MSCs)	Bone Marrow MSCs (BM-MSCs)	Adipose Tissue MSCs (AD-MSCs)
Tissue origin		
Neonatal (extra-embryonic umbilical cord).	Adult tissue (iliac crest/bone marrow); highly sensitive to age/comorbidities.	Adult tissue (subcutaneous fat); sensitive to age/metabolic profile.
Proliferative capacity & doubling time		
Highest. Shortest doubling time, can be expanded to higher passages without entering early senescence.	Lowest. Longest doubling time, prone to replicative senescence at early passages (often around P5–P7).	Moderate to high. Faster growth kinetics than BM-MSCs, but slower than WJ-MSCs.
Preclinically revealed mechanisms: neuroprotection & secretome		
Superior expression of neurotrophic factors (BDNF, NGF, GDNF) and anti-inflammatory cytokines. Enhanced protection against oxidative stress-induced cell death.	Robust secretion of HGF, VEGF, and IGF-1, but overall lower paracrine yield compared to neonatal sources.	High production of VEGF, HGF, and Angiopoietin-1; demonstrates excellent baseline angiogenic support.
Preclinical revealed mechanisms: immunomodulation		
Highest tolerogenicity. Strongest immunosuppressive profile. Express higher baseline levels of HLA-G and lower HLA Class I. Effectively switch microglia from pro-inflammatory M1 to repair M2 phenotypes.	Moderately suppress T-cell proliferation. Interact with late-stage pro-inflammatory signals like HMGB1 and S100 Protein family that facilitate BM-MSCs proliferation.	Strong suppression of T-lymphocyte activation via downregulating CD38 and arresting T-cell cycles. Highly effective at modulating acute peripheral immune responses.
Preclinical stroke models outcomes		
Drastic reduction in infarct volume and profound recovery in sensorimotor functions when administered in both acute and subacute stroke phases, both systemically, directly to the infarcted area, or intranasally.	Consistent reduction in ischemic damage. However, therapeutic impact drops if cells are harvested from aged animals or animals with comorbidity like diabetes.	Superior recovery profiles in focal cerebral ischemia and limb ischemia models compared to autologous adult BM-MSCs, primarily driven by rapid blood–brain barrier stabilization.
Clinical application: harvesting & source logistics		
Completely non-invasive collection from discarded post-birth material. High yield per extraction. Primarily used as an allogeneic product.	Invasive, painful harvest via bone marrow aspiration. Yield declines with donor’s age. Primarily used as autologous source, require time to expand ex vivo.	Minimally invasive harvest via liposuction. Higher starting cell yield vs. BM-MSCs. Frequently evaluated for autologous use.
Current status in clinical trials		
Rising preference for allogeneic stroke therapies due to rapid availability, lower risk of graft-versus-host disease, and high batch consistency.	Most extensively documented in clinical trials, but limitedly used due to slow manufacturing timelines for autologous acute stroke settings.	Increasingly utilized in clinical settings for central nervous system and vascular repair due to abundant donor tissue accessibility.

Note: BDNF—Brain-Derived Neurotrophic Factor; GDNF—Glial Cell Line-Derived Neurotrophic Factor; HGF—Hepatocyte Growth Factor; HLA—Human Leukocyte Antigen; HLA-G—Human Leukocyte Antigen G; HMGB1—High Mobility Group Box 1 Protein; IGF-1—Insulin-Like Growth Factor 1; NGF—Nerve Growth Factor; VEGF—Vascular Endothelial Growth Factor.

## Data Availability

No new data were created or analyzed in this study. Data sharing is not applicable to this article.

## Abbreviations

The following abbreviations are used in this manuscript:

AD-MSC	Adipose Tissue Mesenchymal Stem Cell
AI	Artificial Intelligence
BBB	Blood–Brain Barrier
Bcl-2	B-cell Lymphoma 2 protein
Bcl-xL	B-cell Lymphoma Extra-Large protein
BDNF	Brain-Derived Neurotrophic Factor
BM-MNC	Bone Marrow Mononuclear Cell
BM-MSC	Bone Marrow Mesenchymal Stem Cell
BMP7	Bone Morphogenetic Protein 7
DAMP	Damage-Associated Molecular Pattern
ESC	Embryonic Stem Cells
FGF-2	Fibroblast Growth Factor 2
FIM	Functional Independence Measure
GDNF	Glial Cell Line-Derived Neurotrophic Factor
GFAP	Glial Fiibrillary Acid Protein
GM-CSF	Granulocyte-Macrophage Colony-Stimulating Factor
GMP	Good Manufacturing Practice
HGF	Hepatocyte Growth Factor
HLA	Human Leukocyte Antigen
HLA-G	Human Leukocyte Antigen G
HMGB1	High Mobility Group Box 1 Protein;
IFN-γ	Interferon Gamma
IGF	Insulin-Like Growth Factor
IL	Interleukin
iPSC	Induced Pluripotent Stem Cell
JAK	Janus Kinase
lncRNA	Long Non-Coding RNA
LRR	Leucine Rich Repeat
MAPK	Mitogen-Activated Protein Kinase
MHC	Major Histocompatibility Complex
MIP-1β	Macrophage Inflammatory Protein-1β
miRNA	Micro RNA
MRS	Modified Rankin Scale
MSC	Mesenchymal Stem Cell
NF-κB	Nuclear Factor Kappa-Light-Chain-Enhancer of Activated B Cells
NGF	Nerve Growth Factor
NIHSS	National Institute of Health Stroke Scale
NLR	Nucleotide-Binding Oligomerization Domain-Like Receptor
NLRP3	Pyrin Domain-Containing Protein 3
PDGF-AA	Platelet-Derived Growth Factor-AA
PEBP1	Phosphatidylethanolamine Binding Protein 1
PINK1	PTEN-Induced Kinase 1
ROS	Reactive Oxygen Species
STAT	Signal Transducer and Activator of Transcription
TGF-β	Transforming Growth Factor-Beta
TLR	Toll-Like Receptor
TNF-α	Tumor Necrosis Factor α
VEGF	Vascular Endothelial Growth Factor
UC-MSC	Umbilical Cord Mesenchymal Stem Cell
VR	Virtual Reality
WJ	Wharton’s Jelly
